# HMM-FRAME: accurate protein domain classification for metagenomic sequences containing frameshift errors

**DOI:** 10.1186/1471-2105-12-198

**Published:** 2011-05-24

**Authors:** Yuan Zhang, Yanni Sun

**Affiliations:** 1 Computer Science and Engineering Department, Michigan State University, East Lansing, MI, USA

## Abstract

**Background:**

Protein domain classification is an important step in metagenomic annotation. The state-of-the-art method for protein domain classification is profile HMM-based alignment. However, the relatively high rates of insertions and deletions in homopolymer regions of pyrosequencing reads create frameshifts, causing conventional profile HMM alignment tools to generate alignments with marginal scores. This makes error-containing gene fragments unclassifiable with conventional tools. Thus, there is a need for an accurate domain classification tool that can detect and correct sequencing errors.

**Results:**

We introduce HMM-FRAME, a protein domain classification tool based on an augmented Viterbi algorithm that can incorporate error models from different sequencing platforms. HMM-FRAME corrects sequencing errors and classifies putative gene fragments into domain families. It achieved high error detection sensitivity and specificity in a data set with annotated errors. We applied HMM-FRAME in Targeted Metagenomics and a published metagenomic data set. The results showed that our tool can correct frameshifts in error-containing sequences, generate much longer alignments with significantly smaller E-values, and classify more sequences into their native families.

**Conclusions:**

HMM-FRAME provides a complementary protein domain classification tool to conventional profile HMM-based methods for data sets containing frameshifts. Its current implementation is best used for small-scale metagenomic data sets. The source code of HMM-FRAME can be downloaded at http://www.cse.msu.edu/~zhangy72/hmmframe/ and at https://sourceforge.net/projects/hmm-frame/.

## Background

Culture-independent methods and high-throughput sequencing technologies now enable us to obtain community random genomes (metagenomes) from different habitats such as arctic soils and mammalian gut. Currently, metagenomic annotation focuses on phylogenetic complexity and protein composition analysis. An important component in protein composition analysis is protein domain classification, which classifies a putative protein sequence into annotated domain families and thus aids in functional analysis. Profile HMM-based alignment is the state-of-the-art method for protein domain classification because of its high sensitivity in classifying remote homologs [1]. In conjunction with the Pfam database [2], which contains over 10,000 annotated protein domain families, HMMER [3] can accurately classify query protein sequences into existing domain families. In addition, the latest version of HMMER can achieve comparable speed to BLAST, making it applicable to large-scale metagenomic data sets.

**Figure 1:**
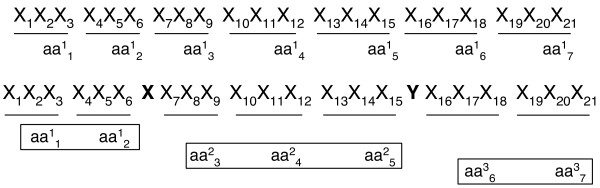
**Frameshifts cause short alignments with marginal scores**. *X_i_*is the *ith *base of a DNA sequence. Every codon is underscored.  is the *jth *amino acid of a peptide sequence derived under reading frame *i*. The correct peptide sequence can be derived from the error-free sequence (shown on the top of the figure) under reading frame 1. Because of insertions of two nucleotides (bolded X and Y), the correct peptide sequence is the concatenation of three short peptide sequences derived using different reading frames. Thus, each peptide sequence derived using one reading frame can only generate short alignments with insignificant scores.

However, HMMER cannot optimally classify sequences containing frameshift errors. In HMMER's domain analysis, six-frame translations of a sequence read or a predicted gene fragment are aligned with annotated protein domain families using HMMER. One problem of this method is that sequencing errors, including insertions or deletions of nucleotides, create frameshifts during translation. As a result, the derived peptide sequences are likely to generate alignments with marginal scores. As HMMER uses alignment scores, E-values, or lengths to determine family membership, these reads become unclassifiable or can be falsely recognized as "novel" proteins during downstream analysis. Figure 1 illustrates how insertion or deletion errors cause marginal alignment scores.

This problem is more serious in domain analysis for metagenomic data sets. Given the high complexity of many metagenomic data sets, high-quality genome assembly is not always available. Thus, protein annotation can only be conducted on short sequence reads. The average read length varies from 25-35 to around 400 bases for the next-generation sequencing methods currently in use. On average there is about one open reading frame per 1000 base pairs in bacteria genomes. Depending on gene size, many gene fragments in metagenomic sequence reads may share only a small overlap with existing domain families, generating even shorter profile HMM alignments with significantly lower scores.

Although a number of tools [4-9] exist for frameshift detection, they are not designed for protein domain classification using profile HMMs. In addition, these tools have not incorporated sequencing error patterns associated with next generation sequencing technologies. A clear disadvantage is that they do not distinguish between error rates in and out of homopolymer regions in pyrosequencing reads. The goal of this work is to design an accurate profile HMM alignment method that can incorporate any given error pattern. Our experiments show that our tool has high sensitivity (>95%) in detecting sequencing errors and has a low false positive rate (~ 0.15%). By correcting insertion and deletion errors, it can generate longer alignments with significantly higher alignment scores, and thus provide more accurate protein domain classification.

## Related work

A number of programs exist to handle frameshifts through DNA versus protein sequence alignment. The simplest methods discard sequences that might contain frameshifts rather than trying to correct them. For example, BLASTX provides insightful information about whether a query DNA sequence contains frameshifts using six-frame translations. However, it neither explicitly outputs positions of insertions or deletions that create frameshifts, nor does it try to fix them by constructing an alignment from pieces obtained from different reading frames. Other tools are available to detect and fix frameshift errors automatically. Frame [4] uses BLASTX to compare all six reading frames of the query nucleotide sequence against protein sequences. Then the aligned regions are combined for frameshift detection. Guan et al. [5], Zhang et al. [6], and Halperin et al. [7] describe dynamic programming algorithms for frameshift detection during pairwise DNA and protein sequence alignment. Instead of using all reading frames of a DNA sequence to maximize the alignment score, another group of tools [8,9] translate a protein sequence back into DNA sequences and formulate the alignment problem as a network matching problem. Frameshift detection has also been applied to finding distant protein homologies where the divergence is the result of frameshift mutations and substitutions [10-12].

Some gene-finding tools detect frameshifts. FrameD [13] relies on a directed acyclic graph for gene prediction in the presence of frameshifts. Kislyuk et al. [14] apply an *ab initio *method to detect possible frameshifts from coding potential generated by GeneMark [15]. GeneTack [16] and FragGeneScan [17] use hidden Markov models for *ab initio *frameshift detection in gene finding.

Despite the extensive study of frameshift detection, the above programs are not designed for protein family classification through DNA versus protein family alignment. Alternatively, Genewise [18], a widely used DNA versus protein alignment tool, allows comparison of a DNA sequence with a profile HMM. Our algorithm differs from Genewise by explicitly incorporating a position-specific error model that is trained on data from different sequencing platforms such as 454 GS FLX Titanium.

## Method

The representative protein domain classification tool HMMER [3] classifies a query protein sequence into a profile HMM-represented protein family using the Viterbi or the Forward algorithm [19]. The Viterbi algorithm aligns a query protein sequence to a profile HMM by searching for the most probable state path in the model. If the alignment score or E-value meets the pre-defined threshold, the query is classified into the corresponding family. The alignment generated by the Viterbi algorithm only accounts for the difference caused by evolutionary divergence between a sequence and a protein family. In order to classify error-containing sequences into their native families, the alignment algorithm must detect the differences resulted from both evolution and sequencing errors.

In this section, we describe HMM-FRAME, the implementation of an augmented Viterbi algorithm that searches for the optimal alignment between a DNA query and a profile HMM by considering both evolutionary divergence and sequencing errors. HMM-FRAME differs from HMMER in the following ways: 1) HMM-FRAME directly accepts a DNA sequence as input, 2) HMM-FRAME accepts a sequencing error model as input, 3) HMM-FRAME can detect and fix frameshifts caused by sequencing errors in the DNA sequence. The output alignment indicates which bases are inserted or deleted due to evolutionary change or sequencing error.

### Error models

Here we describe the error models used in our experiments. Different sequencing technologies may have different types of errors. For example, previous work [20-22] has shown that insertions and deletions occur more often in homopolymer regions than in non-homopolymer regions for pyrosequencing reads. Substitution errors occur more often than insertions or deletions in Illumina sequencing reads. Because deletion or insertion errors cause frameshifts, we focus on applying HMM-FRAME to pyrosequencing data sets.

In this work, we consider two error models. The first one is a published model trained from GS20 sequencing reads [20]. The insertion and deletion error rates in non-homopolymer and homopolymer regions are 0.0007 and 0.0044, respectively. The second error model is computed on data from FLX Titanium sequencing platform. We obtained a set of Titanium sequence reads (Cole and Wang, unpublished) extracted from the region H of the 16S rRNA, which were amplified from the Baylor mock community (22 strains, 24 sequences). Then we computed error rates using insertions and deletions that were annotated by generating careful Needleman-Wunsch alignments between the Titanium sequencing reads and the control sequences. In total, 7,040 sequences passed the initial quality control of RDP [23] after contamination and chimera detection. There were 1,721 insertion and deletion errors. Note that PCR, which was used to generate the amplicons of the sample, can introduce errors. However, because most of the errors introduced by PCR are substitution errors, we assumed that the deletions and insertions were mainly sequencing errors. The derived error rates for homopolymers of different sizes were: 1: 0.000532, 2: 0.000698, 3: 0.00102, 4: 0.000688, 5: 0.0372, 6: 0.00167, 7: 0.143, where the first number is the size of homopolymer regions (1 means non-homopolymer) and the second number is the rate of insertion and deletion errors. If we sum the error rates for homopolymer regions of different sizes, the insertion and deletion error rates for non-homopolymer and homopolymer regions were 0.0005 and 0.001, respectively. They are slightly smaller than the published G20 error rates [20]. We will compare their performance on a data set with annotated errors in the **Results and Discussion **section.

### The augmented Viterbi algorithm for sequencing error correction

Let *π *be a state path in a profile HMM *M *. Let *r *be a set of insertion and deletion positions in a DNA sequence *x*. The augmented Viterbi algorithm searches for the most probable path π* and the most probably error position set r* such that (π*, r* ) = *argmax*_(*π,r*)_*P *(*x, π, r*). Intuitively this algorithm searches for an optimal alignment between a DNA sequence and a profile HMM by simultaneously considering 1) evolutionary divergence (i.e. the insertion, deletion, and substitution of amino acids) and 2) sequencing errors (i.e. insertion and deletion of nucleotides). To solve the above equation, we first divide the search space according to different types of sequencing errors inside a codon and between two consecutive codons. For each type of error, we search for the most probable state path.

**Input**: a DNA sequence *x*, a profile HMM *M *, and a sequencing error model. Notations of *M *and the error model will be described below.

**Output**: the optimal alignment between DNA sequence *x *and *M *, as well as error positions in *r*.

**Algorithm: **we first define notations that will be used in the dynamic programming equations.

• **Notations about the profile HMM ***M*: States *M_j_*, *I_j_*, and *D_j _*are matching, insertion, and deletion states in *M*.  is the transition probability from state *s*_1 _to *s*_2_. *e_s_*(*T *(*x*_*i*-2_*x*_*i*-1_*x*_*i*_)) is the emission probability for state *s *to emit amino acid *T *(*x*_*i*-2_*x*_*i*-1_*x*_*i*_), which is translated from the codon *x*_*i*-2_*x*_*i*-1_*x*_*i*_. For a detailed description of a profile HMM *M *, we refer the reader to the textbook [19] and the users' guide of HMMER [3]. State *G_j _*is the only state that is not defined in profile HMMs from HMMER 3.0. It encodes insertions of nucleotides between codons.  is the transition probability from matching state *M_j _*to nucleotide insertion state *G_j_*. It is set to the insertion error probability.  is the self-transition probability for *G_j_*, encoding the probability of consecutive insertions. When consecutive insertion is not allowed, it is set to 0.  is the transition probability from *G*_*j*-1 _to the next matching state *M_j_*. When only one insertion error is allowed, it is set to 1.0.

• **Notations about the sequencing error model**: *p_I_*(*x_i_*) is the probability that base *x_i_*is an insertion error. *p_D_*(*x_i_*) is the probability that there is a deletion error after base *x_i_*.

• **Subproblems and the recursive equations**: Based on our analysis of error patterns, it is very rare that there are consecutive insertions or deletions in a sequence read. Thus, the following DP algorithm assumes that there is at most one insertion or deletion inside a codon. The algorithm can be extended to handle all possible cases.

-  is the score of the best alignment matching subsequence *x*_1..*i *_to the submodel up to the matching state *M_j_*, given that *x_i_*is the third base of a codon and this codon encodes an amino acid emitted by *M_j_*.

-  is the score of the best alignment matching subsequence *x*_1..*i *_to the submodel up to the insertion state *I_j_*, given that *_T _*(*x*_*i*-2_*x*_*i*-1_*x*_*i*_) is emitted by *I_j_*.

-  is the score of the best alignment ending in *x_i_*being emitted by state *G_j_*, which encodes an insertion of nucleotides between codons.

-  is the score of the best alignment matching subsequence *x*_1..*i *_to the submodel up to the deletion state *D_j_*.

In cases IV and V, we use **d **to represent the deleted bases. We choose **d **to maximize the emission probability of *T *(*x*_*i*-1_**d ***x_i_*) (or *T *(**d ***x*_*i*-1_*x_i_*)) in the matching state *M_j_*.

#### Running time analysis

The time complexity of the above dynamic programming algorithm is *O*(*δ|x||M |*), where *|x| *is the length of input DNA sequence and *|M | *is the number of states in *M *. *δ *is the number of different types of errors inside a codon plus the case of insertions between two codons. In our current implementation, *δ *= 26, which renders a longer running time than the standard Viterbi algorithm. Thus, it is not practical to compare millions of metagenomic sequence reads to over 10,000 protein families in Pfam. Instead, we only run HMM-FRAME on sequences that are likely to contain insertion or deletion errors. For large-scale applications, we suggest applying HMMER 3.0, which is as fast as Blast [24], to all input sequence reads using a big E-value cutoff (such as 100). Alignments covering at least 80% of the translated DNA sequence with significant E-values can be classified by HMMER in this step. Sequence reads that do not yield any partial alignments are unlikely to be members of any protein family. Thus, we only apply HMM-FRAME to reads yielding partial alignment with marginal scores because these reads could potentially contain sequencing errors.

## Results and Discussion

In this section, we compare the sensitivity and false positive rates (FP rates) of HMM-FRAME with Genewise [18] and FragGeneScan [17]. We then apply HMM-FRAME to Targeted Metagenomics and a published metagenomic data set. Our experimental results show that the length, scores, and E-values of profile HMM alignments are significantly improved after error correction. As profile HMM-based alignment tools determine membership by comparing E-value or length with user-defined thresholds, the improvement of these parameters enables more error-containing sequences to be classified into their native families.

### Accuracy of HMM-FRAME

In order to evaluate the accuracy of HMM-FRAME in detecting insertion and deletion errors, we obtained a control data set with annotated error positions from RDP (Cole and Wang, unpublished). In this data set, NifH gene families from the *Desulfitobacterium hafniense *strain DCB-2, the *Burkholderia xenovorans *strain LB40, and the PCC 7120 strain of *Anabaena *were amplified and then sequenced using 454 Titanium. The sequenced gene families were aligned with the nifH genes in these three organisms using the Needleman-Wunsch algorithm. Insertion and deletion errors were identified from the alignments. After contamination and chimera screening, we had 18,900 sequences, of which 3,408 sequences contained 4,623 insertion or deletion errors. We conducted the protein domain analysis on the 18,900 sequences using HMM-FRAME under the two error models presented in the **Method **Section. The input profile HMM was trained on 25 nifH genes obtained from RDP's functional gene repository website [25].

We evaluated the performance of error-prediction tools using two types of sensitivity and FP rates. Let *S*^+ ^be the set of error-containing sequences in the control data set. Let *S *be the set of predicted error-containing sequences. The *Sequence-level sensitivity *and *FP rate *are  and , respectively. Similarly, let *Q*^+ ^be the set of insertion and deletion positions in error-containing sequences from the control data set. Let *Q *be the set of predicted error positions. The *Base-level sensitivity *and *FP rate *are  and , respectively.

Using the control data set, we first evaluated the performance of HMM-FRAME under the published GS20 and our self-trained Titanium error models. Then we compared the performance of HMM-FRAME with Genewise [18] and FragGeneScan [17]. Similar to HMM-FRAME, Genewise can directly compare DNA sequences with a profile HMM and can accept user-defined error rates. We tested Genewise using different parameters including error rates and the alignment score thresholds (ranging from 0 to 20). The results with the best tradeoff between sensitivity and FP rate were kept for comparison with HMM-FRAME. FragGeneScan [17] is a newly developed gene prediction tool for short and error-prone sequences. It predicts genes and identifies sequencing errors inside predicted genes. We applied FragGeneScan on the above sequence set (all genes) and tested its sensitivity and FP rate. FragGeneScan successfully recognized all input as protein-coding genes, rendering a high gene-prediction sensitivity in this data set. However, FragGeneScan had higher FP rates than HMM-FRAME in error detection. The results are summarized in Table 1.

**Table 1:** Comparing the error detection performance of HMM-FRAME, Genewise, and FragGeneScan

	HMM-FRAME: G20	HMM-FRAME: self-trained	GeneWise	FragGeneScan
seq-sen	**95.25%**	90.6%	53.8%	83.04%
base-sen	**85.08%**	82.4%		53.39%
seq-FP	0.154%	**0**	0.001%	0.7%
base-FP	2.1%	**0.003%**		59.57%

Sensitivity and FP rate of each program when detecting annotated insertion and deletion errors in nifH genes. seq-sen: sequence-level sensitivity. base-sen: base-level sensitivity. seq-FP: sequence-level FP rate. base-FP: base-level FP rate. The score cutoff of Genewise is set to zero to maximize the sensitivity. As Genewise has low sequence-level sensitivity, we did not evaluate its performance at the base-level.

As shown in Table 1, each tool has higher sensitivity and smaller FP rates in identifying error-containing sequences than in locating error positions. HMM-FRAME has a better tradeoff between sensitivity and FP rate than both Genewise and FragGeneScan. Both GS20 and our self-trained Titanium error models have small FP rates in predicting error positions, but GS20 has higher sensitivity. Thus, we plan to use GS20 in all further experiments.

### Using HMM-FRAME in "Targeted Metagenomics"

In this section, we present the utility of HMM-FRAME in two applications of "Targeted Metagenomics", where one or several gene families are amplified from environmental DNA and these amplicons are sequenced using high-throughput sequencing platforms. One typical application of Targeted Metagenomics is to sequence the amplicons of the 16S rRNA gene for phylogenetic complexity analysis. Besides 16S rRNA, protein-coding genes that are important to a particular habitat can be amplified and sequenced for targeted functional analysis in metagenomic data sets. For example, Targeted Metagenomics of the nifH gene, which encodes nitrogenase reductase, is important for analyzing microbial genomes sequenced from soil. Although these sequences are sampled from one or several targeted gene families, frameshift errors can cause short alignments with marginal scores between the input and the targeted gene families. As a result, sequences lacking significant alignment length and scores will be regarded as contaminants and be discarded. Thus, it is desirable to fix frameshift errors to maximize the number of usable samples. Given a DNA read and a profile HMM built from a set of known protein sequences, HMM-FRAME can be applied to detect and correct frameshift errors in amplicon reads.

In the first experiment, we obtained 3,937 nifH sequences of an average length of 76 bases generated by the 454 FLX sequencing technology. In order to discard contaminants that originated from non-target genes, we aligned the 3,937 sequences with the nifH gene family, which was built on a small set of 25 expert-verified full-length nifH protein reference sequences from RDP's functional gene repository [25]. In the gene family building process, we first applied ClustalW [26] to align the 25 reference sequences. Then we applied HMMER 3.0's hmmbuild program to derive a profile HMM from the multiple sequence alignment. Of the 3,937 454 FLX sequences, 111 were found to be contaminants and were excluded from further analysis. Of the remaining 3,826 sequences, HMM-FRAME detected 296 insertions and deletions in 256 sequences. Thus, approximately, 7% of the samples contained frameshift errors. Of the 256 sequences containing insertion or deletion errors, 224 (87.5%) only contained one insertion or deletion error. 24 (9.4%) sequences contained two errors, and eight (3.1%) contained three errors. Of the 296 insertions or deletions, 224 (75.7%) were inside or beside homopolymer regions.

Because protein domain classification tools compare alignment lengths, scores, and E-values with pre-defined thresholds to determine a sequence's membership, the changes in the alignments affect the final domain composition analysis. After error correction, profile HMM-based alignment tools are expected to generate longer alignments with bigger scores and smaller E-values. This gives error-containing sequences a better chance of being classified into the correct families rather than being labeled contaminants.

**Figure 2:**
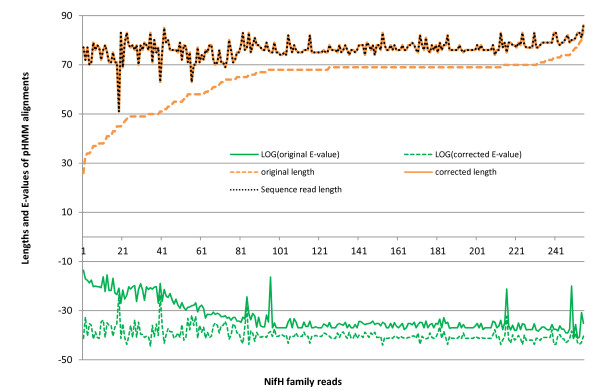
**Change of HMMER alignments' scores, lengths, and E-values (in log space) before and after error correction for nifH sequences**. HMMER 3.0 alignments of sequences before and after error correction by HMM-FRAME. The changes of alignments are presented for 256 sequences in which HMM-FRAME detects errors. "Original" refers to HMMER 3.0 alignments on sequences before error correction. "Corrected" refers to HMMER 3.0 alignments on sequences after error correction by HMM-FRAME. As a comparison, we also plot the length of the original sequence reads (with the legend "sequence read"). They largely overlap with the length of corrected alignments, indicating that complete sequence reads can be aligned with the nifH profile HMM after error correction.

In order to conduct a fair comparison on alignments before and after error correction, we choose a third-party tool HMMER 3.0 to generate alignments for original and corrected sequences. The changes of alignments' E-values and lengths due to error correction are presented in Figure 2. In order to test whether the improvement was statistically significant, we conducted a two-sample Kolmogorov-Smirnov test (K-S test) on the alignments' lengths and E-values before and after error correction. The p-values for the alignments' length and E-value distributions were 3.1037e-010 and 1.1802e-045, respectively. In particular, the comparison between alignments' lengths and the sequence reads' lengths shows that most partial alignments generated by error-containing sequences become complete alignments after error correction. Thus, when comparatively longer alignments (e.g., 23 amino acids or 69 bases) are required for domain classification, more sequence reads (213 more under when the threshold is 69 bases) will be classified into their native families.

### Protein domain analysis of the bacterial aromatic dioxygenase genes

**Figure 3:**
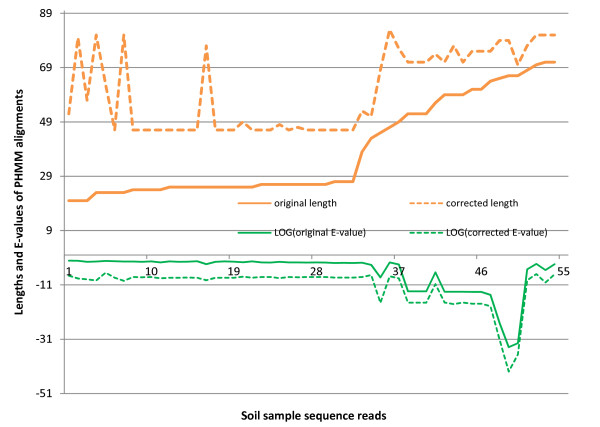
**Change of HMMER alignments' lengths, scores, and E-values (in log space) before and after error correction for the bacterial aromatic dioxygenase genes in a soil sample**. The data set are sequenced from bacterial aromatic dioxygenase genes in a soil sample. All alignments are generated by HMMER 3.0 for a fair comparison. "Original" refers to HMMER 3.0 alignments on sequences before error correction. "Corrected" refers to HMMER 3.0 alignments on sequences after error correction by HMM-FRAME.

In the second experiment, we obtained 2486 pyrosequencing samples of an average length of 224 bases from the bacterial aromatic dioxygenase genes in a soil sample [27]. Although these pyrosequencing reads were sequenced from the 5' end of PCR amplicons of bacterial aromatic dioxygenase genes, we were interested in classifying them into three sub-families of dioxygenase genes: toluene/biphenyl, naphthalene, and benzoate [28]. Note that there is another subfamily (phthalate). However, due to lack of training proteins for this family (Dr. Iwai, personal communication), we only searched for members of three sub-families. Three sets of reference protein sequences were extracted from Pfam [2] for toluene/biphenyl, naphthalene, and benzoate [28]. Based on these training sets, we built three profile HMMs using ClustalW and HMMER 3.0. Then we applied HMM-FRAME to align the 2486 reads with the three profile HMMs. HMM-FRAME detected 77 insertions and 52 deletions, which were distributed in 121 sequences. Of the 121 error-containing sequences, 77 could not be classified into any subfamily by HMMER 3.0 under the E-value threshold 0.1. After error correction using HMM-FRAME, these 77 sequences were classified into different families with an average E-value of 3.3e-06, indicating that they were highly likely to be true members of the underlying families. For other error-containing sequences, the profile HMM alignments' E-values and lengths were significantly increased after error correction. The change is plotted in Figure 3. We applied a two-sample K-S test on the alignments' lengths and E-values before and after error correction. The p-values for the length and E-value distributions were 8.0609e-011and 1.9776e-040, respectively. The improved alignment lengths and E-values provide stronger evidence for the membership of the input samples. In total, after error correction by HMM-FRAME, we could classify 1,214 sequences into three subfamilies. 1,042 reads were members of the naphthalene subfamily. 96 reads belonged to the benzoate subfamily. 76 reads belonged to the toluene/biphenyl subfamily. The remaining 1272 reads could potentially be members of the subfamily phthalate (Dr. Iwai, personal communication).

### Protein domain classification in the deep mine data set

In order to show the utility of HMM-FRAME in a metagenomic data set containing members of multiple domain families, we applied HMM-FRAME to the first 454 sequencing project for environment samples, which were sequenced from two sites in the Soudan Mine, Minnesota, USA [29]. In this experiment, we downloaded the Black Sample from the paper's supplementary data website. This data set contains 388,627 sequence reads with an average length of 99 bases.

**Figure 4:**
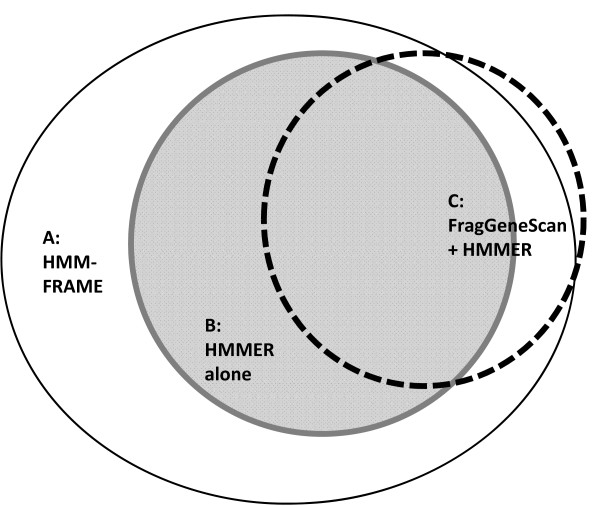
**Protein domain classification results for the black sample in the deep mine data set**. Sequence sets that can be classified by HMM-FRAME, HMMER, and FragGeneScan+HMMER are represented by three sets A, B, and C. *|A| *= 17,496. *|B| *= 13,544. *|C| *= 12,328. B-C = 2224. C-B = 1008. C-A = 4. A-(B+C) = 2948.

There were two steps in the annotation. First, we applied gene-prediction tools. Second, we conducted the domain classification on predicted genes. A number of gene-prediction tools are available for metagenomic data sets. However, not every tool can handle short reads. Glimmer [30] did not output meaningful predictions when it was applied to this data set. The sensitivity of Metagene [31] drops to 59% for 100-base sequences [32]. We thus chose FragGeneScan, a newly developed gene-prediction tool for short reads. FragGeneScan predicted 281,658 genes, of which 72,355 contained errors. For convenience in discussion, let S be the set of genes predicted by FragGeneScan. Let S' be the raw read set corresponding to genes in S. Thus, 72,355 sequences in S were different from their raw reads in S' because FragGeneScan predicted and corrected errors in S'. We compared three domain classification pipelines: 1) apply HMMER 3.0 on raw reads S', 2) apply FragGeneScan and then HMMER 3.0 on corrected reads S, and 3) apply HMM-FRAME on raw reads S'. We recorded how many reads could be classified into one of the 2,558 Pfam domain families that contain the keyword "bacteria". The number of classifiable reads for the three pipelines were: 13,544 for HMMER, 12,328 for FragGeneScan + HMMER, and 17,496 for HMM-FRAME. The classification results have large overlaps, which are illustrated in Figure 4. In summary, HMM-FRAME was able to classify 2,948 more reads than the other two annotation pipelines. HMM-FRAME found errors in all of these 2,948 reads. Thus, it is likely that other two pipelines failed to classify them because of frameshifts. HMM-FRAME failed to classify four reads that can be aligned by FrageGeneScan + HMMER. A closer examination showed that FragGeneScan and HMM-FRAME output different error positions in these four sequences.

The performance evaluation of FragGeneScan must consider both gene-prediction and error-prediction. Of the 281,658 predicted genes, only 12,328 could be classified into existing domain families. Further analysis is needed to examine whether other predictions are novel genes or wrong predictions. It is worth noting that FragGeneScan could classify 1,008 more sequences after its error correction than applying HMMER 3.0 alone on raw reads. However, while 2,224 raw reads could be classified into existing domain families by HMMER 3.0, they could not be aligned with any family after error correction by FragGeneScan. This indicates that FragGeneScan might have over-predicted errors in the 2,224 sequences. This is consistent with our observation that FragGeneScan has a high FP rate in the control data set.

## Conclusion and future directions

Despite the advances of high-throughput sequencing technologies, sequencing errors still pose challenges for data annotation. In particular, our error model analysis shows that 454 FLX Titanium only slightly decreases the insertion and deletion error rates compared to GS20. Thus, correcting frameshifts caused by insertion or deletion errors is still important for metagenomic sequence annotation. In this work, we introduce a protein domain classification tool HMM-FRAME, which can classify error-prone DNA sequence reads into protein domain families. HMM-FRAME can accept any error model trained on data from high-throughput sequencing technologies and thus achieve high detection sensitivity while maintaining a low false positive rate.

Applying HMM-FRAME to a data set with annotated errors shows its high sensitivity and accuracy in error detection. In particular, by fixing frameshift errors, we can obtain significantly longer profile HMM alignments with smaller E-values. As alignments' lengths, scores, and E-values are often used to determine family membership, improving them helps to classify more sequences into the native domain families. In our experiments, sequences that fail HMMER 3.0 under the default E-value or score threshold are classified into correct domain families using HMM-FRAME. Thus, HMM-FRAME can be used as a complementary tool to HMMER 3.0 on error-prone sequences.

HMM-FRAME is more computationally expensive than HMMER 3.0 mainly because of diverse sequencing errors inside codons. We plan to improve the efficiency of our DNA versus profile HMM alignment algorithm so that it can be used efficiently in large-scale protein domain analysis. Besides applying DP matrix pruning techniques to reduce the computational cost, we plan to use a faster but less accurate Viterbi algorithm as a filtration stage. Specifically, we can apply a faster Viterbi algorithm to predict whether there are any errors inside a codon before identifying error positions. If such errors exist, we can then use a more sensitive method to determine the exact number and positions of insertions or deletions.

## Authors' contributions

YS designed the algorithm, experiments, and wrote the manuscript. YZ implemented the algorithm and conducted the experiments. Both authors read and approved the final manuscript.
